# Supplementary material for: Enhancing the Antibody Production Efficiency of Chinese Hamster Ovary Cells through Improvement of Disulfide Bond Folding Ability and Apoptosis Resistance
Source: Cells. 2024 Sep 4;13(17):1481. doi: 10.3390/cells13171481 (PMC11394227; doi:10.3390/cells13171481)
Supplement: Supplementary file 1 [file cells-13-01481-s001.zip › cells-3111015-supplementary.pdf]

Title: Enhancing the antibody production efficiency of CHO cells through improvement of disulfide bond folding ability and apoptosis resistance

Authors: Chen Zhang[1], Yunhui Fu[1], Wenyun Zheng[2], Feng Chang[1], Yue Shen[1], Jinping Niu[1], Yangmin Wang, and Xingyuan Ma[1*]

Affiliations:

1 State Key Laboratory of Bioreactor Engineering, School of Biotechnology, East China University of Science and Technology, Shanghai, China

2 Shanghai Key Laboratory of New Drug Design, School of Pharmacy, East China University of Science and Technology, Shanghai, China

* Correspondence: maxy@ecust.edu.cn

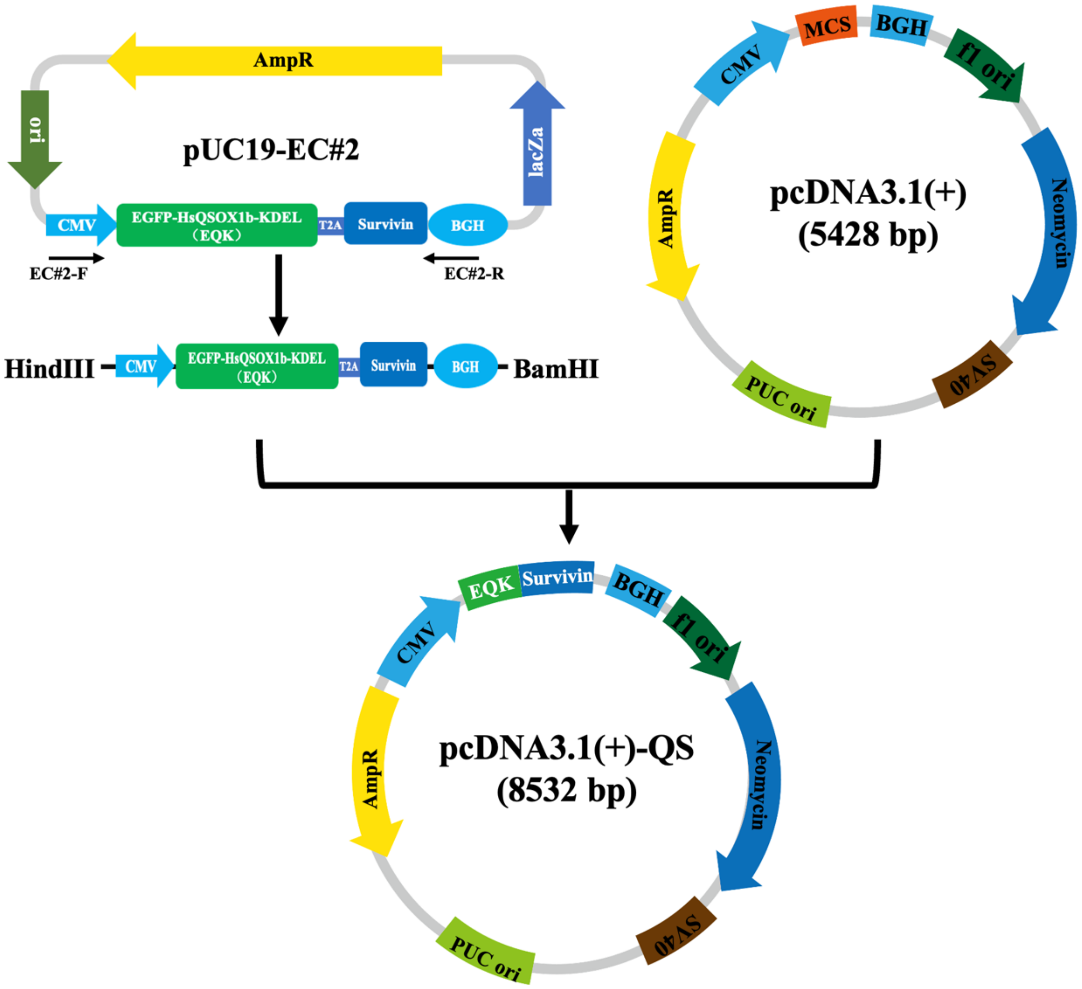

Figure S1. pcDNA3.1 (+)-QS construction process diagram.

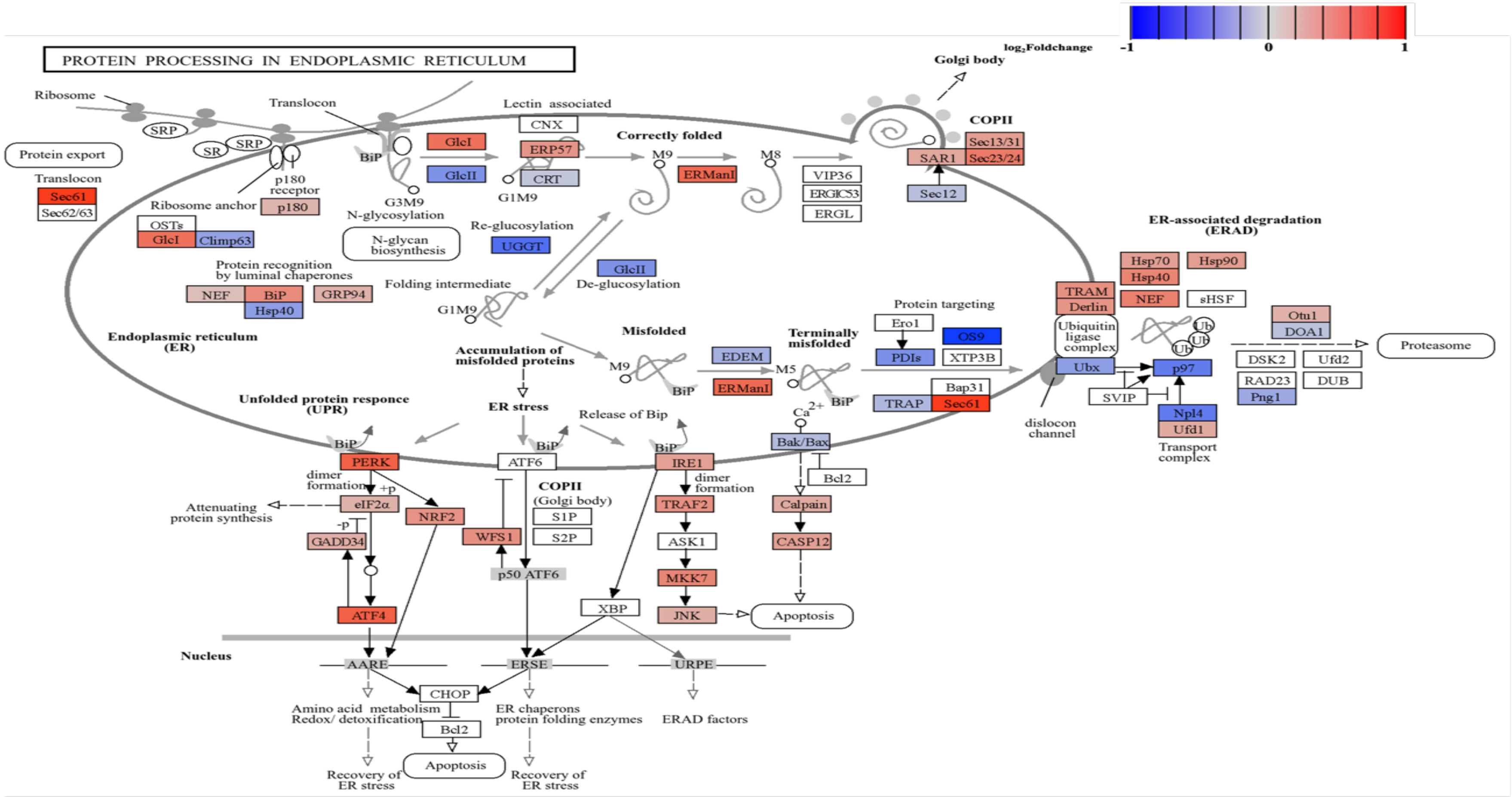

Figure S2. KEGG pathway of CHO-PAb-QS protein processing pathway in the ER (map04141). The genes are color-coded according to their log2Foldchange values (p-value < 0.05), where white-marked genes are p-value ≥ 0.05 or genes that have not been sequenced.

Table S1. List of differentially expressed genes in the KEGG pathway.

Gene_id	Symbol	$Log_2FoldChange$	p-value	Regulation	Pathway
Atf4	Atf4	0.672282702	8.71E-25	Up	UPR
Bak1	Bak	-0.16862121	0.00371137	Down	UPR
Calr	CRT	-0.096321606	0.02018275	Down	UPR
Capn2	Calpain	0.244984785	5.70E-07	Up	UPR
Ckap4	Climp63	-0.316584499	2.29E-10	Down	UPR
Derl2	Derlin	0.372010608	0.00185249	Up	ERAD
Dnajc10	HSP40	-0.345212036	5.56E-09	Down	Protein processing
Dnaja1	HSP40	0.461786651	7.10E-12	Up	UPR
Edem1	EDEM	-0.236072075	0.03417859	Down	Protein processing
Eif2ak3	PERK	0.666347281	1.58E-13	Up	UPR
Eif2s1	eIF2α	0.207314384	1.32E-21	Up	UPR
Ern1	IRE1	0.309547898	0.00105131	Up	UPR
Ganab	Glc II	-0.07227941	7.31E-26	Down	Protein processing
Hsp90aa1	Hsp90	0.318383405	7.51E-10	Up	ERAD
Hspa5	Bip	0.419142614	5.91E-22	Up	UPR
Hspa8	Hsp70	0.30462382	1.06E-10	Up	ERAD
Hsp90b1	GRP94	0.20755582	4.78E-07	Up	Protein processing
Hsph1	NEF	0.482490626	4.51E-18	Up	ERAD
Hyou1	NEF	0.135395856	0.00693999	Up	Protein processing
LOC100759746	Casp 12	1.40006891	4.15E-26	Up	UPR
Man1c1	ERManI	0.61264725	1.10E-11	Up	Protein processing
Map2k7	MKK7	0.4586149	4.38E-08	Up	Nfe2l2
Mapk8	JNK	0.22428507	0.01268588	Up	UPR
Mogs	GlcI	0.5721843	4.82E-06	Up	Protein processing
Nfe2l2	NRF2	0.3792281	2.30E-10	Up	UPR
Ngly1	Png1	-0.2815813	0.01379662	Down	ERAD
Nploc4	Npl4	-0.5593097	3.48E-26	Down	ERAD
Nsfl1c	Ubx	0.61423537	1.94E-22	Up	ERAD
Os9	Os9	-0.9379257	1.41E-48	Down	ERAD
P4hb	PDIs	-0.4291328	7.06E-22	Down	Protein processing
Pdia3	ERP57	0.36907147	3.88E-13	Up	Protein processing
Plaa	DOA1	-0.1287218	0.04005155	Down	ERAD

Ppp1r15a	GADD3	0.22868138	0.02077037	Up	UPR
Preb	Sec12	-0.5770717	7.90E-10	Down	Protein processing
Prkcsh	GlcII	-0.4184271	2.47E-14	Down	Protein processing
Rrbp1	p180	0.18636531	0.00675827	Up	Protein processing
Sar1b	SAR1	0.26004829	0.02448585	Up	Protein processing
Sec13	Sec13/31	0.3115414	5.25E-06	Up	Protein processing
Sec23a	Sec23/24	0.5150848	4.02E-12	Up	Protein processing
Sec61a2	Sec61	0.87116529	6.45E-21	Up	Protein processing
Ssr4	TRAP	-0.1861326	0.02800721	Down	ERAD
Traf2	TRAF2	0.18623148	0.01523358	Up	UPR
Tram1	TRAM	0.39441679	1.04E-05	Up	ERAD
Ubxn8	UBx	-0.3434072	9.28E-05	Down	ERAD
Ufd1	Ufd1	0.2464084	0.00011895	Up	ERAD
Uggt2	UGGT	-0.6331415	7.03E-11	Down	Protein processing
Vcp	p97	-0.5492256	1.27E-28	Down	ERAD
Wfs1	WFS1	0.39693138	5.03E-13	Up	UPR
Yod1	Otu1	0.21057865	1.37E-05	Up	ERAD
Bet1l	Bet1	0.20672831	0.0461814	Up	Vesicular transport
Gosr1	Gos1	-0.2629951	0.00068533	Down	Vesicular transport
Gosr2	Bos1	0.46013255	1.19E-06	Up	Vesicular transport
Stx17	Stx17	0.83106266	5.44E-40	Up	Vesicular transport
Sec22b	Sec22	0.51050131	0.00066283	Up	Vesicular transport
Caspase-3	Casp3	-0.2176117	0.040665875	Down	Apoptosis
Caspase-7	Casp7	-0.1904572	0.01403847	Down	Apoptosis
Caspase-8	Casp8	-0.3465352	5.34E-05	Down	Apoptosis
Fadd	FADD	-0.4372268	0.00241247	Down	Apoptosis
LOC100757098	Cytc	0.18233965	0.04286191	Up	Apoptosis
Tnfrsf1a	TNFR	-0.7179568	8.25E-24	Down	Apoptosis
Xiap	XIAP	0.57232183	0.04302619	Up	Apoptosis
